# Predictors of urinary incontinence in community-dwelling frail older adults with diabetes mellitus in a cross-sectional study

**DOI:** 10.1186/1471-2318-14-137

**Published:** 2014-12-16

**Authors:** Amy Hsu, Jessamyn Conell-Price, Irena Stijacic Cenzer, Catherine Eng, Alison J Huang, Kathy Rice-Trumble, Sei J Lee

**Affiliations:** VA Quality Scholars Fellow, Geriatrics and Extended Care, San Francisco VA Medical Center, 4150 Clement Street, 181G, San Francisco, CA 94122 USA; Division of Geriatrics, University of California at San Francisco, San Francisco, CA USA; University of California at San Francisco, San Francisco, CA USA; Geriatrics and Extended Care, San Francisco VA Medical Center, San Francisco, CA USA; On Lok Lifeways, San Francisco, CA USA; Division of General Internal Medicine, University of California at San Francisco, San Francisco, CA USA; VA Quality Scholars Fellowship Senior Scholar, Geriatrics and Extended Care, San Francisco VA Medical Center, San Francisco, CA USA

**Keywords:** Urinary incontinence, Frail older adults, Diabetes mellitus

## Abstract

**Background:**

Diabetes mellitus is a potent risk factor for urinary incontinence. Previous studies of incontinence in patients with diabetes have focused on younger, healthier patients. Our objective was to characterize risk factors for urinary incontinence among frail older adults with diabetes mellitus in a real-world clinical setting.

**Methods:**

We performed a cross-sectional analysis on enrollees at On Lok (the original Program for All-Inclusive Care of the Elderly) between October 2004 and December 2010. Enrollees were community-dwelling, nursing home-eligible older adults with diabetes mellitus (N = 447). Our outcome was urinary incontinence measures (n = 2602) assessed every 6 months as “never incontinent”, “seldom incontinent” (occurring less than once per week), or “often incontinent” (occurring more than once per week). Urinary incontinence was dichotomized (“never” versus “seldom” and “often” incontinent). We performed multivariate mixed effects logistic regression analysis with demographic (age, gender and ethnicity), geriatric (dependence on others for ambulation or transferring; cognitive impairment), diabetes-related factors (hemoglobin A1c level; use of insulin and other glucose-lowering medications; presence of renal, ophthalmologic, neurological and peripheral vascular complications), depressive symptoms and diuretic use.

**Results:**

The majority of participants were 75 years or older (72%), Asian (65%) and female (66%). Demographic factors independently associated with incontinence included older age (OR for age >85, 3.13, 95% CI: 2.15-4.56; Reference: Age <75) and African American or other race (OR 2.12, 95% CI: 1.14-3.93; Reference: Asian). Geriatric factors included: dependence on others for ambulation (OR 1.48, 95% CI: 1.19-1.84) and transferring (OR 2.02, 95% CI: 1.58-2.58) and being cognitively impaired (OR 1.41, 95% CI: 1.15-1.73). Diabetes-related factors associated included use of insulin (OR 2.62, 95% CI: 1.67-4.13) and oral glucose-lowering agents (OR 1.81, 95% CI: 1.33-2.45). Urinary incontinence was not associated with gender, hemoglobin A1c level or depressive symptoms.

**Conclusions:**

Geriatric factors such as the inability to ambulate or transfer independently are important predictors of urinary incontinence among frail older adults with diabetes mellitus. Clinicians should address mobility and cognitive impairment as much as diabetes-related factors in their assessment of urinary incontinence in this population.

## Background

Urinary incontinence (UI) is common among frail older adults [1–4] and is associated with substantial morbidity and mortality. UI significantly decreases quality of life [5–8], increases the risk of depression [5, 9], disability [5, 6], social isolation [6], loss of dignity [8] and poor self-rated health [5, 10]. Further, it is associated with increases in adverse outcomes, including falls [11, 12], fractures [11], hospitalization [13], nursing home admission [13] and has been linked with mortality [14].

Diabetes is a potent risk factor for UI, increasing both the prevalence [15–19] and severity [20] of UI. Previous research suggests that 10 – 50% of older adults with diabetes experience UI [21–23]. Middle-aged and older ambulatory women with diabetes mellitus have increased odds of having UI compared to women without diabetes [16–20]. Women with diabetes mellitus were also twice as likely to develop more severe UI with enough leakage to wet outer clothing compared to women without diabetes [20]. Although frail community-dwelling older adults with diabetes represent a large, growing population at high risk for UI, to our knowledge, no studies have examined the risk factors for UI in this population. Thus, we sought to characterize risk factors for UI among nursing home-eligible, community dwelling frail older adults with diabetes mellitus in a real-world setting.

## Methods

### Participants

**Figure 1:**
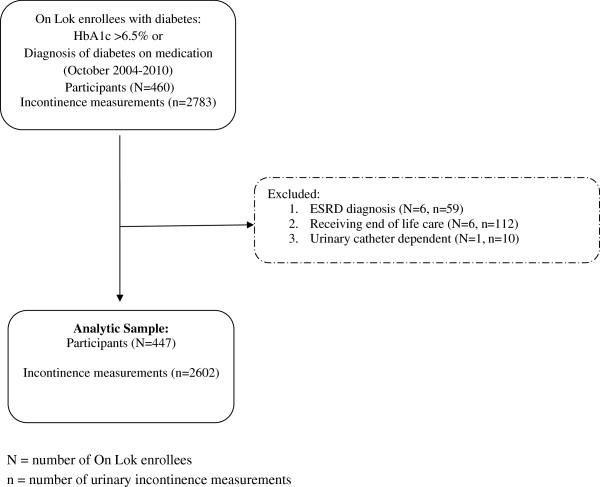
**Inclusion and exclusion criteria for participants and measurements; ESRD = End** **-stage renal disease.**

We studied all On Lok enrollees diagnosed with diabetes mellitus between October 2004 and December 2010 (N = 447 participants with n = 2602 UI measurements). On Lok, the original model for Programs for All-inclusive Care for the Elderly (PACE), requires enrollees to be nursing home-eligible, indicating that the participant requires care with full-time supervision of a licensed nurse. On Lok helps nursing home-eligible enrollees remain in the community by providing and coordinating healthcare services, including primary and specialist physician services, adult day health care, home care, hospital care, post-acute rehabilitation care and custodial nursing home care. On Lok provides enrollees with transportation between home and PACE centers where meals, medication management, help with bathing or showering and recreational activities are provided. Further, On Lok centers have physical and occupational therapists, social workers, nurses and physicians on-site. Each enrollee receives a comprehensive health assessment (medical evaluation with assessment of function and geriatric syndromes) upon enrollment and every 6 months thereafter by physicians, nurses, therapists and social workers.Enrollees were eligible for our study if they were enrolled in On Lok during the study period and had a diagnosis of diabetes mellitus on a glucose lowering medication or a hemoglobin A1c (HbA1c) level greater than 6.5% (Figure 1). Diabetes diagnosis was determined according to the International Classification of Diseases, Ninth Revision (ICD-9) code, 250.xx.

We initially identified 460 (N) On Lok enrollees with 2783 (n) UI measurements. UI measurements were excluded from the analysis if enrollees had end-stage renal disease on dialysis (N = 6, n = 59), were receiving end-of-life care (N = 6, n = 112) or had a urinary catheter (N = 1, n = 10). These exclusion criteria led to our final analytic cohort of 447 enrollees with 2602 UI measurements. All data were extracted from electronic medical records. The Committee on Human Research at the University of California San Francisco and the San Francisco VA Research and Development Committee approved this study. Further, they found that this study satisfied federal criteria for waiving informed consent since 1) it poses minimal risks to participants, 2) it would not be practicable to obtain informed consent and 3) participants rights are not adversely affected by waiving informed consent.

To capture the dynamic nature of diabetes and UI in this population, our unit of analysis was measures of urinary incontinence. Thus, a patient who was in our study for 9 months would contribute two UI measurements: the initial admission assessment and the subsequent 6 month follow-up assessment. This allowed us to explore whether risk factors that change over time such as insulin use or HbA1c levels affect incontinence.

### Measures: outcome

The primary outcome was urinary incontinence, evaluated during semi-annual comprehensive health assessments. A nurse or therapist determined the presence and frequency of UI by conducting interviews with enrollees and corroborating with family caregivers and home care aides. Nurses and therapists were trained to code UI occurring less than once a week as “seldom incontinent” and UI occurring more than once per week as “often incontinent”.

We compared measures of “never incontinent” to the combined measure of “seldom incontinent” and “often incontinent”. To explore whether some factors were associated with more severe incontinence, we also compared “never incontinent” to “often incontinent”.

### Measures: potential predictors

We examined a wide range of potential factors that may be associated with urinary incontinence. Specifically, we examined demographic factors including age, gender and race/ethnicity. We examined geriatric factors including dependence in ambulation, dependence in transferring and cognitive impairment. Ambulation and transferring was assessed by a nurse who made an in-person determination of whether the individual was independent, required supervision, required assistance, was dependent on others or non-ambulatory. All levels except for independent were categorized as “dependent.” Participants with a Mental Status Questionnaire (MSQ) score greater than 4 were considered to have cognitive impairment. Participants without MSQ scores but had an ICD-9 diagnosis of dementia were also considered to have cognitive impairment.

We examined diabetes-related factors including the use of glucose lowering medications, HbA1c levels, and diabetes-related complications such as renal or ophthalmologic complications, peripheral vascular disease, and neurological disease (through ICD-9 codes). To determine the HbA1c level on the day of urinary incontinence assessment, we interpolated HbA1c values, assuming that the HbA1c changes in a linear fashion between 2 measured values. For example, if the first measured HbA1c value is 7.0% and the next measured value 100 days later is 8.0%, the interpolated HbA1c value is 7.1% on Day 10, 7.2% on Day 20, etc. We also examined whether depressive symptoms and diuretic use was associated with urinary incontinence. Presence of depressive symptoms was defined by a short Geriatric Depression Scale score greater than 9.

Several other factors were considered, but were not included in the final analysis due to a low number of participants and measurements (less than 10%) with these risk factors. These included: obesity, benign prostatic hypertrophy, prostate cancer (determined using ICD-9 codes) and use of a urinary antispasmodic agent.

### Statistical analysis

Subjects with and without incontinence were characterized using descriptive bivariate statistics. We used ANOVA to compare the means of continuous variables (age and HbA1c level) and Chi-square tests to compare categorical variables. We performed multivariate analyses to identify independent risk factors for urinary incontinence using mixed effects logistic regression to account for clustering of incontinence measurements by participant. We adjusted for age, gender, Asian race, dependence in transferring and ambulating, cognitive impairment, use of thiazide or loop diuretics, depression, diabetic medication use, and diabetic complications (renal, ophthalmologic, peripheral vascular, and neurological). All analyses were performed using Stata MP (version 10.1, StataCorp, College Station, TX) and SAS (version 9.2, SAS System of Windows, SAS Institute Inc., Cary, NC).

## Results

### Characteristics of the participants

Table 1 shows the characteristics of the participants at initial assessment by level of urinary incontinence. The overall prevalence of UI was 44% at baseline. The majority of participants were 75 years or older with a high proportion of female and Asian enrollees. Participants had an average of 5 assessments (range 1–19). At the first assessment 56% were “never incontinent”, 28% were “seldom incontinent”, and 16% were “often incontinent”. Participants were frail older adults with complex medical issues. Forty-two percent were dependent with ambulation and 36% had cognitive impairment. Severe urinary incontinence was more common at baseline in older adults with dependence in ambulation and transferring and cognitive impairment. Diuretic use was common in our study population at 25%. Depressive symptoms were present in 25% of participants. Depressive symptoms were present in 57% of those with measurements of “often incontinent” but in fewer than 30% of those with “never incontinent” or “seldom incontinent” measures. Participants had appropriately controlled diabetes according the American Geriatrics Society guidelines [24], with 70% of participants with HbA1c of less than 8% at the first visit within our study. However, forty-eight percent of participants had renal complications related to diabetes while other diabetes-related complications were less common.

**Table 1:** **Baseline characteristics of participants by urinary incontinence status, N = 447**

Characteristic	Number of persons, N	Never incontinent, N	Seldom incontinent, N	Often incontinent, N	p-value
**Total persons**	447	249 (56%)	125 (28%)	73 (16%)	
**Mean Age**		78 (±8.2)	80 (±8.1)	81 (±8.0)	0.961
**Age categories**					
<75	127	88 (70%)	27 (21%)	12 (9%)	0.003
75-80	101	52 (52%)	34 (34%)	15 (15%)	
80-85	118	64 (54%)	28 (24%)	26 (22%)	
>85	101	45 (56%)	36 (36%)	20 (16%)	
**Race/Ethnicity**					
Asian	290	168 (58%)	73 (25%)	49 (17%)	0.364
White	58	25 (43%)	21 (36%)	12 (21%)	
African	35	19 (54%)	11 (31%)	5 (15%)	
American/Other					
Latino	64	37 (58%)	20 (31%)	7 (11%)	
**Gender**					
Male	154	98 (64%)	40 (26%)	16 (10%)	0.018
Female	293	151 (51%)	85 (29%)	57 (19%)	
**Ambulation** ^**a**^					
Independent	254	173 (68%)	61 (24%)	20 (8%)	0.000
Dependent^b^	188	75 (40%)	63 (34%)	50 (27%)	
**Transferring** ^**c**^					
Independent	317	204 (64%)	82 (26%)	31 (10%)	0.000
Dependent^b^	127	45 (35%)	42 (33%)	40 (32%)	
**Cognitive Impairment**					
Absent	228	184 (64%)	71 (25%)	33 (11%)	0.000
Present	159	65 (41%)	54 (34%)	40 (25%)	
**Other Medications**					
**Loop or Thiazide Diuretic Use**					
No	336	188 (56%)	91 (27%)	57 (17%)	0.679
Yes	111	61 (55%)	34 (31%)	16 (14%)	
**Other factors**					
**Depressive** ^**d**^ **Symptoms**					
Absent	329	187 (57%)	87 (26%)	55 (17%)	0.287
Present	110	59 (54%)	37 (34%)	14 (13%)	
**Hemoglobin A1c Level**					
**Mean (95% CI)**		7.7 (7.5-7.9)	7.6 (7.3-7.8)	7.7 (7.4-8.0)	0.645
<7	163	87 (53%)	53 (33%)	23 (14%)	0.382
7-7.9	152	82 (54%)	42 (28%)	28 (18%)	
8-8.9	69	46 (67%)	13 (19%)	10 (14%)	
≥9	63	34 (54%)	17 (27%)	12 (19%)	
**Diabetes Medications**					
None	272	157 (58%)	70 (38%)	45 (17%)	0.527
Oral Medications	133	73 (55%)	39 (29%)	21 (16%)	
Insulin	42	19 (45%)	16 (38%)	7 (17%)	
**Diabetes Complications**					
**Renal**					
Absent	200	106 (53%)	57 (29%)	37 (19%)	0.460
Present	247	143 (58%)	68 (28%)	36 (15%)	
**Ophthalmologic**					
Absent	299	165 (55%)	80 (27%)	54 (18%)	0.338
Present	148	84 (57%)	45 (30%)	19 (13%)	
**Peripheral vascular**					
Absent	384	216 (56%)	107 (28%)	61 (16%)	0.784
Present	63	33 (52%)	18 (29%)	12 (19%)	
**Neurological**					
Absent	336	180 (54%)	103 (31%)	53 (16%)	0.088
Present	111	69 (62%)	22 (20%)	20 (18%)	

^a^N = 5 with missing observations.

^b^Partially or fully dependent.

^c^N = 3 with missing observations.

^d^N = 6 with missing observations.

### Independent predictors of incontinence

Many non-diabetes factors were strongly associated with urinary incontinence (Table 2). Demographic factors (increasing age and African American or other and White race) were associated with urinary incontinence. Although female gender was associated with incontinence in the bivariate analysis, gender was not an independent predictor after adjustment. Geriatric factors were also strongly associated with urinary incontinence. Partial or full dependence with ambulation or transferring were both associated with urinary incontinence with the odds ratio for transferring remaining over 2 after adjustment. Two factors commonly associated with urinary incontinence, diuretic use and presence of depressive symptoms, were not associated with urinary incontinence in this study.

**Table 2:** **Multivariate analysis of factors associated with urinary incontinence**

Characteristic	Never vs. Seldom/often incontinent		Never vs. often incontinent	
	Unadjusted OR (95% CI)	Adjusted ^a^OR (95% CI)	Unadjusted OR (95% CI)	Adjusted ^a^OR (95% CI)
Non-diabetes related factors				
**Demographics**				
**Age**				
<75	1.00 (Reference)	1.00 (Reference)	1.00 (Reference)	1.00 (Reference)
75-80	1.79 (1.37-2.35)	1.57 (1.17-2.11)	1.38 (0.94-2.01)	1.18 (0.73-1.89)
80-85	2.61 (1.92-3.56)	2.11 (1.51-2.95)	2.84 (1.87-4.31)	2.35 (1.44-3.84)
>85	4.75 (3.36-6.71)	3.13 (2.15-4.56)	3.76 (2.38-5.93)	2.29 (1.34-3.92)
**Race/Ethnicity**				
Asian	1.00 (Reference)	1.00 (Reference)	1.00 (Reference)	1.00 (Reference)
White	1.59 (0.99-2.55)	2.15 (1.28-3.59)	1.67 (0.93-3.01)	1.80 (0.93-3.48)
African	1.29 (0.73-2.28)	2.12 (1.14-3.93)	1.76 (0.90-3.44)	2.43 (1.16-5.08)
American/Other				
Latino	0.99 (0.64-1.53)	1.08 (0.67-1.75)	0.94 (0.51-1.70)	0.91 (0.49-1.70)
**Gender**				
Male	1.00 (Reference)	1.00 (Reference)	1.00 (Reference)	1.00 (Reference)
Female	1.55 (1.13-2.14)	1.27 (0.91-1.78)	1.38 (0.90-2.11)	0.95 (0.61-1.49)
**Geriatric factors**				
**Ambulation**				
Independent	1.00 (Reference)	1.00(Reference)	1.00 (Reference)	1.00 (Reference)
Dependent^b^	2.34 (1.98-2.76)	1.48 (1.19-1.84)	2.89 (2.31-3.61)	1.92 (1.37-2.67)
**Transferring**				
Independent	1.00 (Reference)	1.00 (Reference)	1.00 (Reference)	1.00 (Reference)
Dependent^b^	2.84 (2.35-3.44)	2.02 (1.58-2.58)	3.72 (2.92-4.75)	2.78 (1.99-3.89)
**Cognitive Impairment**				
Absent	1.00 (Reference)	1.00 (Reference)	1.00 (Reference)	1.00 (Reference)
Present	1.67 (1.40-1.99)	1.41 (1.15-1.73)	1.71 (1.37-2.13)	1.85 (1.39-2.46)
**Other Medications**				
**Loop or Thiazide Diuretic**				
No	1.00 (Reference)	1.00 (Reference)	1.00 (Reference)	1.00 (Reference)
Yes	1.24 (0.93-1.65)	0.81 (0.57-1.15)	0.94 (0.66-1.36)	0.64 (0.39-1.04)
**Other factors**				
**Depressive Symptoms**				
Absent	1.00 (Reference)	1.00 (Reference)	1.00 (Reference)	1.00 (Reference)
Present	0.91 (0.64-1.29)	0.97 (0.67-1.41)	0.78 (0.48-1.25)	0.75 (0.45-1.23)
**Diabetes-related factors**				
**Hemoglobin A1c Level**				
<7	1.05 (0.89-1.23)	1.01 (0.84-1.22)	0.86 (0.71-1.05)	0.75 (0.57-1.00)
7-7.9^c^	1.00 (Reference)	1.00 (Reference)	1.00 (Reference)	1.00 (Reference)
8-8.9	0.76 (0.63-0.91)	0.81 (0.65-1.01)	0.86 (0.69-1.08)	0.94 (0.69-1.29)
≥9	0.69 (0.55-0.87)	0.85 (0.65-1.11)	0.85 (0.64-1.12)	1.10 (0.74-1.63)
**Diabetes Medications**				
None	1.00 (Reference)	1.00 (Reference)	1.00 (Reference)	1.00 (Reference)
Oral Medications	1.66 (1.30-2.13)	1.81 (1.33-2.45)	1.50 (1.11-2.03)	1.79 (1.17-2.75)
Insulin	2.30 (1.58-3.37)	2.62 (1.67-4.13)	2.33 (1.53-3.54)	3.41 (1.88-6.21)
**Diabetes Complications**				
**Renal**				
Absent	1.00 (Reference)	1.00 (Reference)	1.00 (Reference)	1.00 (Reference)
Present	0.93 (0.68-1.26)	0.95 (0.68-1.33)	0.76 (0.51-1.13)	0.73 (0.46-1.14)
**Ophthalmologic**				
Absent	1.00 (Reference)	1.00 (Reference)	1.00 (Reference)	1.00 (Reference)
Present	0.82 (0.60-1.13)	1.00 (0.71-1.43)	0.70 (0.46-1.09)	0.68 (0.42-1.10)
**Peripheral vascular**				
Absent	1.00 (Reference)	1.00 (Reference)	1.00 (Reference)	1.00 (Reference)
Present	1.68 (1.08-2.61)	1.31 (0.81-2.10)	2.30 (1.38-3.83)	2.33 (1.35-4.02)
**Neurological**				
Absent	1.00 (Reference)	1.00 (Reference)	1.00 (Reference)	1.00 (Reference)
Present	0.72 (0.51-1.02)	0.73 (0.49-1.08)	1.05 (0.68-1.65)	1.43 (0.87-2.36)

^a^Adjusted for age, gender, Asian race, depression, cognitive impairment, use of thiazide or loop diuretics, diabetic medication use, diabetic complications (neurological, renal, ophthalmologic, peripheral vascular, other), and dependence in transferring and/or ambulating.

^b^Partially or fully dependent.

^c^Hemoglobin A1c level of 7% to 7.9% was used as the reference since the AGS “Guideline for improving the care of older persons with diabetes mellitus” recommends <8% as appropriate target for glycemic control for “frail elders with limited life expectancy [24]”.

Several diabetes-related factors in the model were significantly associated with urinary incontinence. The strongest association was the use of insulin followed by use of oral glucose-lowering agents. HbA1c level was not found to be associated with increased odds of UI.

The sensitivity analysis performed by comparing “never incontinent” with “often incontinent” showed similar results. However, peripheral vascular disease was found to be independently associated with being “often incontinent” (OR 2.33; 95% CI: 1.35, 4.02) but not when “seldom” was combined with “often incontinent” (OR 1.31; 95% CI: 0.81, 2.10). A second sensitivity analysis performed using ordinal logistic regression with 3 outcome categories (never, seldom and often incontinent) showed that dependence in ambulation (OR 1.61; 95% CI: 1.14-2.27), transferring (OR 3.32; 95% CI: 2.28-4.84) and presence of cognitive impairment (OR 1.73; 95% CI: 1.29-2.32) continued to be statistically significantly correlated with urinary incontinence. The differences were that the age group of 75–80 years old (OR 1.34; 95% CI: 0.86-2.1), White race (OR 1.54; 95% CI: 0.93-2.58), the use of oral glucose-lowering agents (OR 1.21, 95% CI: 0.8-1.82) and insulin (OR 1.48; 95% CI: 0.81-2.68) were no longer statistically significantly associated with urinary incontinence. Having peripheral vascular disease was found to be statistically significant (OR 1.89, 95% CI: 1.1-3.26).

## Discussion

Among community-dwelling, nursing home-eligible frail older adults with diabetes mellitus, we found that risk factors common to all older adults were as important as diabetes-related factors in predicting UI. Specifically we found that older age, dependence on others for ambulation or transferring and cognitive impairment were all independent predictors for urinary incontinence. In contrast, among the diabetes-related factors we examined, only the use of diabetes medications were independent risk factors for urinary incontinence.

We found that three factors that are often invoked as potentially important risk factors were not associated with UI among our cohort of nursing home-eligible, community-dwelling older adults. First, diuretic use has been proposed as a risk factor since the use of these medications may increase the volume of urine and worsen UI [25] and the association may be specific to those with uninhibited detrusor contractions [26]. We did not detect a significant association between diuretic use and UI after adjustment for other potential risk factors. Previous studies that found an association between diuretic use and UI focused on younger women [25] or on those using high doses of loop diuretics [27]. Thus, our findings suggest that the type and dose of diuretic may be important factors in the diuretic-UI association.

Second, poor glycemic control has been proposed as a risk factor, since hyperglycemia can lead to both glycosuria and neuropathy, both of which may exacerbate UI [28–30]. We found no association between HbA1c level and UI. Previous studies have also shown a lack of association between HbA1c level and UI [31, 32]. Our results suggest that improving glycemic control to HbA1c < 9% may not lead to substantial improvements in UI. Since both HbA1c levels and diuretic use were not associated with UI, urine volume may not be as important a mechanism for UI in frail, community-dwelling older adults with diabetes.

Lastly, our study did not find an association between UI and depressive symptoms. Two reasons may account for the absence of an association. First, prior studies have been performed in different populations and used different tool to measure depressive symptoms [5]. Second, depression may be mediated by functional limitations, which other studies have not controlled for [9]. Improvement in physical performance has also been associated with lower rates of incident UI [33].

Most previous research on UI in older adults has either focused on nursing home residents or healthier, ambulatory older adults residing in the community. Research among the nursing home population has shown high prevalence of UI from 65% [6] to 70% [3] and increased odds of UI in African Americans [3]. Among older adults residing in the community, prior research has shown that the risk of UI increases with age [1, 5], impaired mobility [1, 34], greater disability [5, 35] and depressive symptoms [5, 9]. Although PACE enrollees reside in the community, they are nursing home-eligible and have a high burden of comorbid medical conditions and functional limitations. Thus, PACE enrollees represent an intermediate population between community dwelling older adults and nursing home residents. Our results reflect this, with an overall prevalence of 44% that is between previous estimates of community-dwelling and nursing home residents.

Few previous studies have focused on UI in community-dwelling, nursing home-eligible populations with diabetes. Khatusky and colleagues examined UI in a general PACE population but relied on annual enrollee survey data to measure incontinence, rather than using clinical assessments [7]. While there are many studies that show that diabetes mellitus is an important risk factor for UI in adult populations [16–18, 20, 34], our study shows that in a frail older adult, PACE enrolled population, factors that affect all frail older adults, such as functional limitations, may be as important in assessing UI as diabetes-related factors.

Our study benefits from the inclusion of community-dwelling frail, older adults with diabetes mellitus who are eligible for nursing home admission and the evaluation of a wide array of both geriatric and diabetes-related factors. However, several limitations should be considered when interpreting our results. First, our UI measurement was based on clinician assessment and categorized together all enrollees with at least weekly UI. Thus we were not able to distinguish specific factors that may lead to severe UI that occurs more frequently than daily. However, clinician assessment reflects real-world evaluation of UI where objective measurements are not often practical or possible. Second, we did not examine participants’ cognitive status with cognitive testing, but used diagnoses of cognitive impairment made by clinicians. However, On Lok clinicians care for many cognitively impaired older adults [36] and are likely to be more experienced in diagnosing cognitive impairment than most clinicians. Third, due to low prevalence of obesity, benign prostatic hypertrophy, prostate cancer and use of urinary antispasmodic agents, we have limited power to detect potential confounding by these factors in our study. Lastly, our study consisted of a large proportion of participants of Asian ethnicity, which may not be generalizable to all community-dwelling nursing home-eligible older adults.

## Conclusions

In conclusion, our findings highlight the complex nature of UI in frail older adults with diabetes. Like all geriatric syndromes, UI is rarely due to a single disease process; rather, it is the result of multiple factors. Thus, clinicians caring for frail older adults with diabetes should address mobility and cognitive impairment as much as glycemic control when managing UI. Further studies testing the relative efficacy of interventions that target UI risk factors are needed to determine how best to manage UI in this vulnerable population.

## Abbreviations

UI: Urinary incontinence
PACE: Programs for all-inclusive care for the elderly
HbA1c: Hemoglobin A1c
MSQ: Mental status questionnaire
ICD-9: International classification of diseases, ninth revision.
